# Supplementary material for: Upregulation of the Long Noncoding RNA CASC10 Promotes Cisplatin Resistance in High-Grade Serous Ovarian Cancer
Source: Int J Mol Sci. 2022 Jul 13;23(14):7737. doi: 10.3390/ijms23147737 (PMC9318856; doi:10.3390/ijms23147737)
Supplement: Supplementary file 1 [file ijms-23-07737-s001.zip › ijms-1796821-supplementary.pdf]

Supplementary materials

Upregulation of the Long Noncoding RNA CASC10 Promotes Cisplatin Resistance in High-Grade Serous Ovarian Cancer

Ricardo Noriega-Rivera [1,2], Mariela Rivera-Serrano [2,4], Robert Rabelo-Fernandez [2,4], Josué Pérez-Santiago [2,3], Fatima Valiyeva [2], and Pablo E. Vivas-Mejía [1,2]*

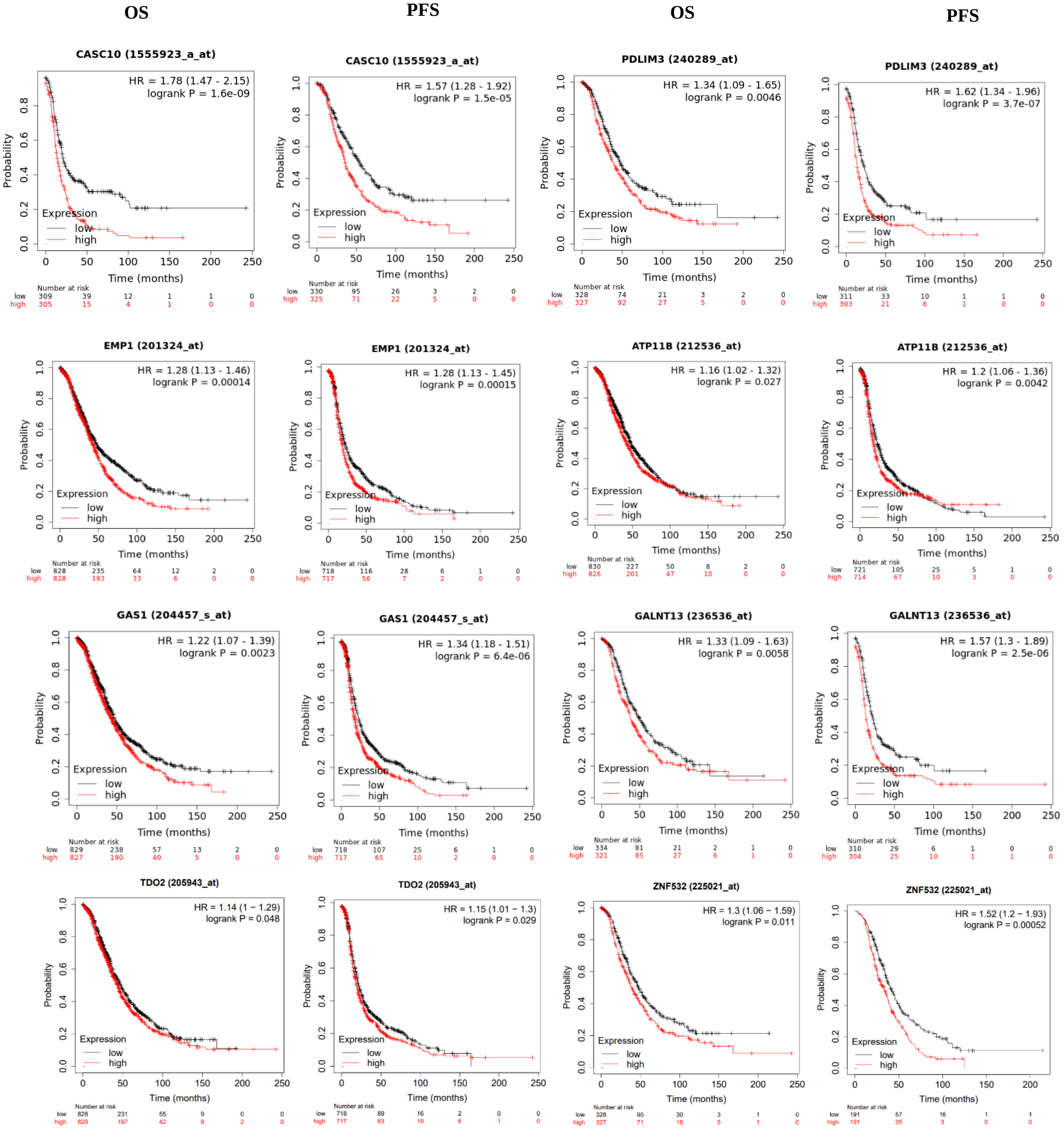

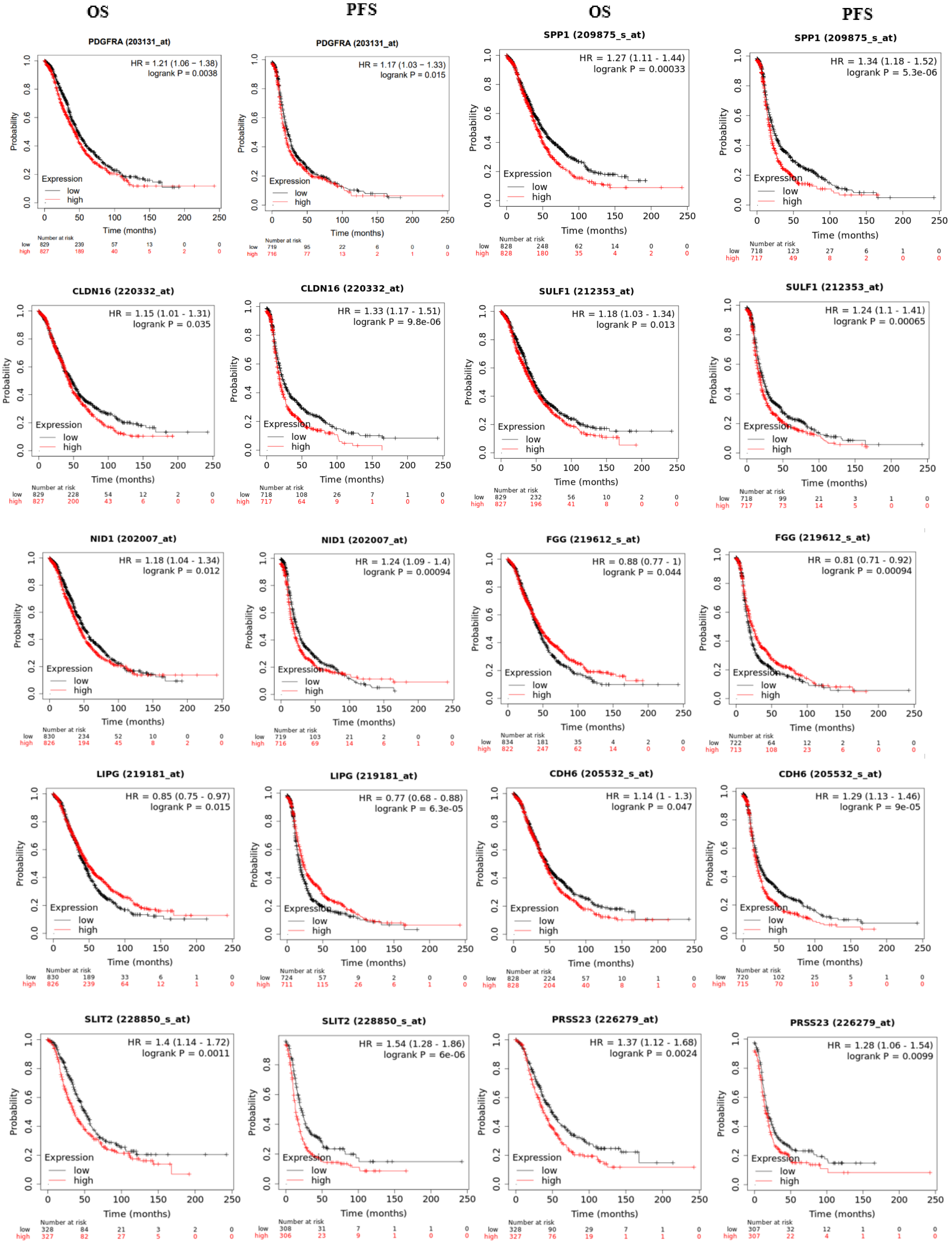

OS
PFS
OS
PFS
PDGFRA (203131_at)
HR = 1.21 (1.06 − 1.38)
logrank P = 0.0038
PDGFRA (203131_at)
HR = 1.17 (1.03 − 1.33)
logrank P = 0.015
SPP1 (209875_s_at)
HR = 1.27 (1.11 - 1.44)
logrank P = 0.00033
SPP1 (209875_s_at)
HR = 1.34 (1.18 - 1.52)
logrank P = 5.3e-06
CLDN16 (220332_at)
HR = 1.15 (1.01 - 1.31)
logrank P = 0.035
CLDN16 (220332_at)
HR = 1.33 (1.17 - 1.51)
logrank P = 9.8e-06
SULF1 (212353_at)
HR = 1.18 (1.03 - 1.34)
logrank P = 0.013
SULF1 (212353_at)
HR = 1.24 (1.1 - 1.41)
logrank P = 0.00065
NID1 (202007_at)
HR = 1.18 (1.04 - 1.34)
logrank P = 0.012
NID1 (202007_at)
HR = 1.24 (1.09 - 1.4)
logrank P = 0.00094
FGG (219612_s_at)
HR = 0.88 (0.77 - 1)
logrank P = 0.044
FGG (219612_s_at)
HR = 0.81 (0.71 - 0.92)
logrank P = 0.00094
LIPG (219181_at)
HR = 0.85 (0.75 - 0.97)
logrank P = 0.015
LIPG (219181_at)
HR = 0.77 (0.68 - 0.88)
logrank P = 6.3e-05
CDH6 (205532_s_at)
HR = 1.14 (1 - 1.3)
logrank P = 0.047
CDH6 (205532_s_at)
HR = 1.29 (1.13 - 1.46)
logrank P = 9e-05
SLIT2 (228850_s_at)
HR = 1.4 (1.14 - 1.72)
logrank P = 0.0011
SLIT2 (228850_s_at)
HR = 1.54 (1.28 - 1.86)
logrank P = 6e-06
PRSS23 (226279_at)
HR = 1.37 (1.12 - 1.68)
logrank P = 0.0024
PRSS23 (226279_at)
HR = 1.28 (1.06 - 1.54)
logrank P = 0.0099
Expression
low
high
Probability
Time (months)
Number at risk

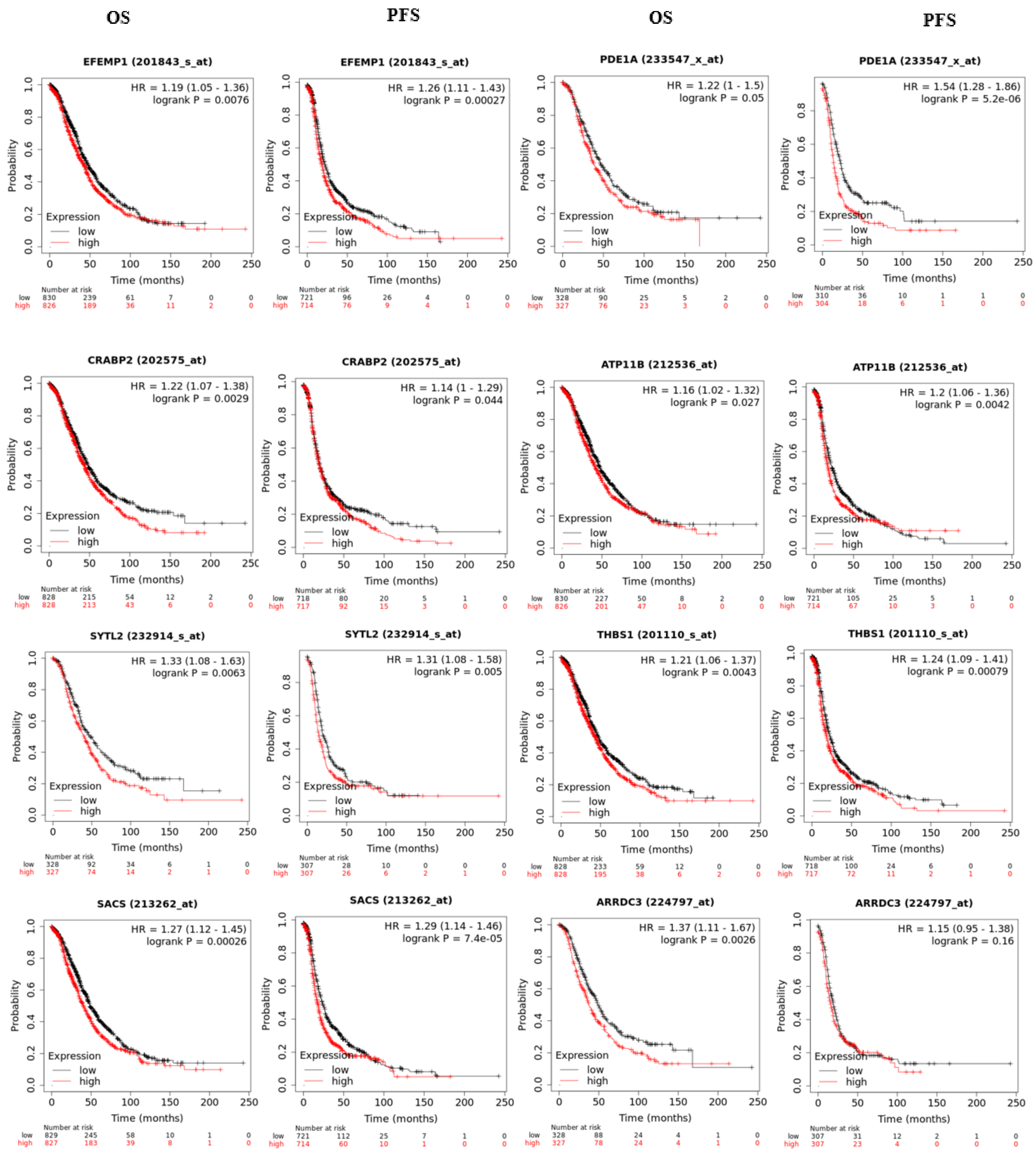
OS
PFS
OS
PFS
EFEMP1 (201843_s_at)
HR = 1.19 (1.05 - 1.36)
logrank P = 0.0076
EFEMP1 (201843_s_at)
HR = 1.26 (1.11 - 1.43)
logrank P = 0.00027
PDE1A (233547_x_at)
HR = 1.22 (1 - 1.5)
logrank P = 0.05
PDE1A (233547_x_at)
HR = 1.54 (1.28 - 1.86)
logrank P = 5.2e-06
CRABP2 (202575_at)
HR = 1.22 (1.07 - 1.38)
logrank P = 0.0029
CRABP2 (202575_at)
HR = 1.14 (1 - 1.29)
logrank P = 0.044
ATP11B (212536_at)
HR = 1.16 (1.02 - 1.32)
logrank P = 0.027
ATP11B (212536_at)
HR = 1.2 (1.06 - 1.36)
logrank P = 0.0042
SYTL2 (232914_s_at)
HR = 1.33 (1.08 - 1.63)
logrank P = 0.0063
SYTL2 (232914_s_at)
HR = 1.31 (1.08 - 1.58)
logrank P = 0.005
THBS1 (201110_s_at)
HR = 1.21 (1.06 - 1.37)
logrank P = 0.0043
THBS1 (201110_s_at)
HR = 1.24 (1.09 - 1.41)
logrank P = 0.00079
SACS (213262_at)
HR = 1.27 (1.12 - 1.45)
logrank P = 0.00026
SACS (213262_at)
HR = 1.29 (1.14 - 1.46)
logrank P = 7.4e-05
ARRDC3 (224797_at)
HR = 1.37 (1.11 - 1.67)
logrank P = 0.0026
ARRDC3 (224797_at)
HR = 1.15 (0.95 - 1.38)
logrank P = 0.16
Expression
low
high
Probability
Time (months)
Number at risk

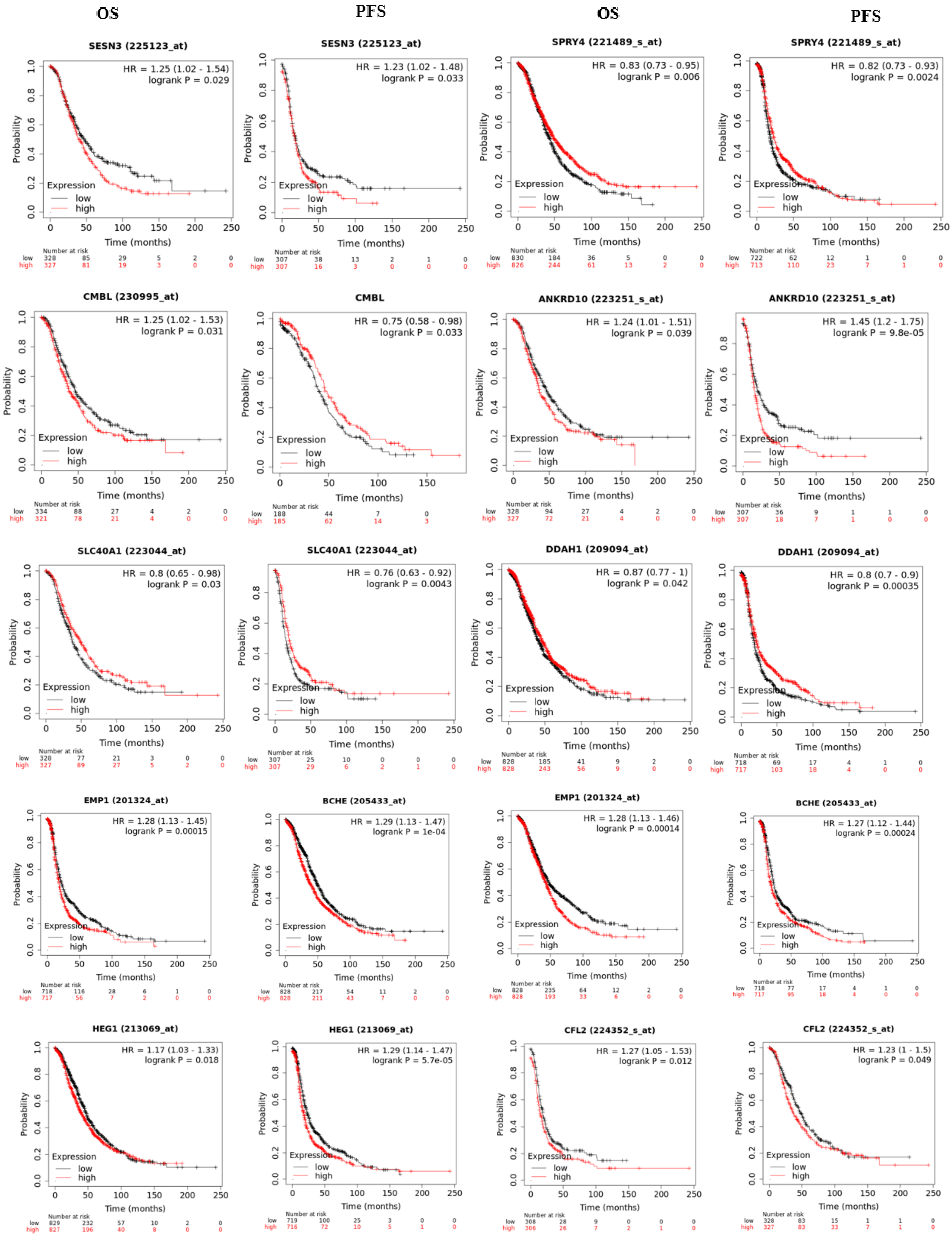

OS
PFS
OS
PFS
SESN3 (225123_at)
HR = 1.25 (1.02 - 1.54)
logrank P = 0.029
SESN3 (225123_at)
HR = 1.23 (1.02 - 1.48)
logrank P = 0.033
SPRY4 (221489_s_at)
HR = 0.83 (0.73 - 0.95)
logrank P = 0.006
SPRY4 (221489_s_at)
HR = 0.82 (0.73 - 0.93)
logrank P = 0.0024
CMBL (230995_at)
HR = 1.25 (1.02 - 1.53)
logrank P = 0.031
CMBL
HR = 0.75 (0.58 - 0.98)
logrank P = 0.033
ANKRD10 (223251_s_at)
HR = 1.24 (1.01 - 1.51)
logrank P = 0.039
ANKRD10 (223251_s_at)
HR = 1.45 (1.2 - 1.75)
logrank P = 9.8e-05
SLC40A1 (223044_at)
HR = 0.8 (0.65 - 0.98)
logrank P = 0.03
SLC40A1 (223044_at)
HR = 0.76 (0.63 - 0.92)
logrank P = 0.0043
DDAH1 (209094_at)
HR = 0.87 (0.77 - 1)
logrank P = 0.042
DDAH1 (209094_at)
HR = 0.8 (0.7 - 0.9)
logrank P = 0.00035
EMP1 (201324_at)
HR = 1.28 (1.13 - 1.45)
logrank P = 0.00015
BCHE (205433_at)
HR = 1.29 (1.13 - 1.47)
logrank P = 1e-04
EMP1 (201324_at)
HR = 1.28 (1.13 - 1.46)
logrank P = 0.00014
BCHE (205433_at)
HR = 1.27 (1.12 - 1.44)
logrank P = 0.00024
HEG1 (213069_at)
HR = 1.17 (1.03 - 1.33)
logrank P = 0.018
HEG1 (213069_at)
HR = 1.29 (1.14 - 1.47)
logrank P = 5.7e-05
CFL2 (224352_s_at)
HR = 1.27 (1.05 - 1.53)
logrank P = 0.012
CFL2 (224352_s_at)
HR = 1.23 (1 - 1.5)
logrank P = 0.049

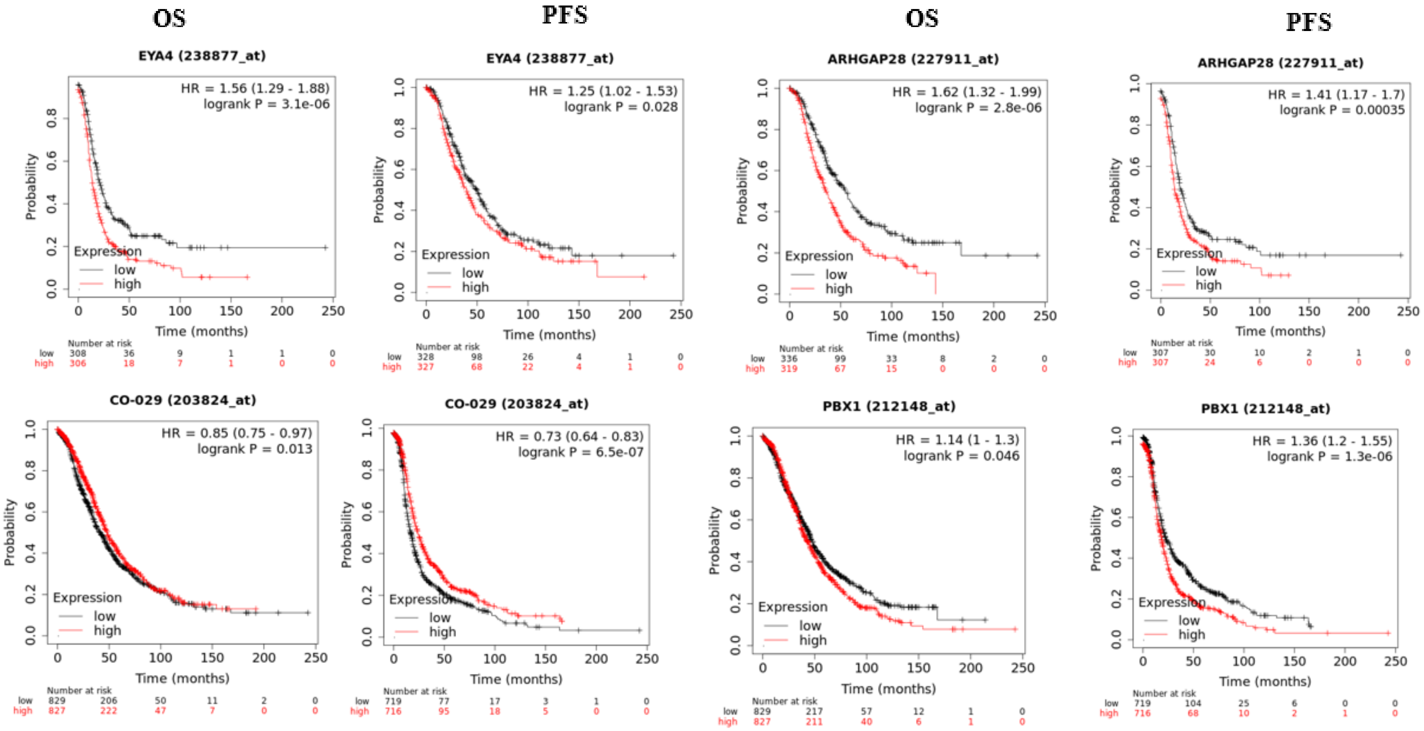

Figure S1. Kaplan–Meier (KM) plots for gene expression-based overall survival (OS) and progression-free survival (PFS) analysis. KM plots of ovarian cancer patients were generated using the KM plotter searchable database. The OS and PFS of patients with ovarian cancer are stratified by expression levels of the additional 57 clinically relevant genes in ovarian cancer. P-values < 0.05 were considered to be statistically significant.

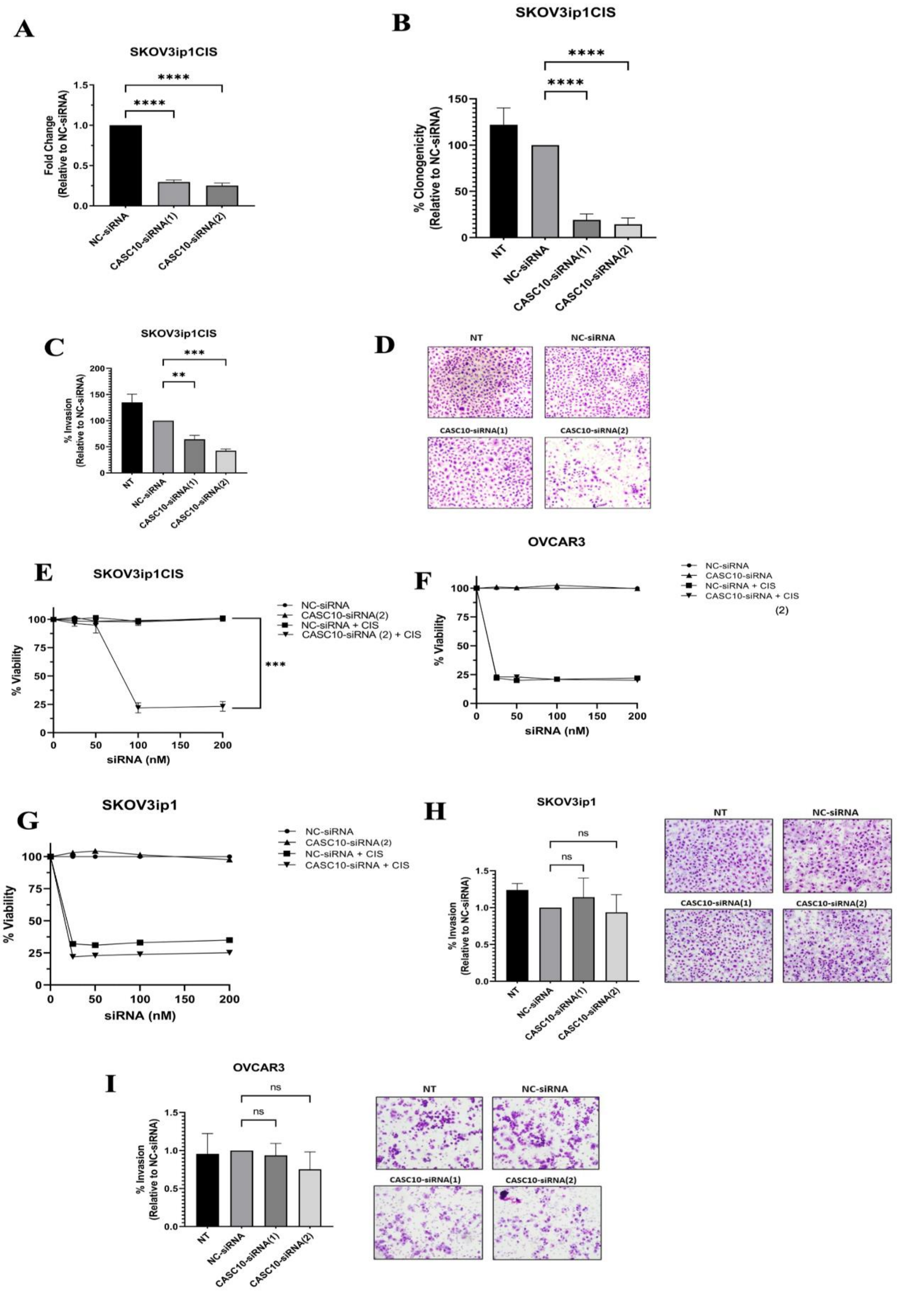

Figure S2. siRNA-mediated CASC10 knockdown reduced cell growth, invasion, and viability in SKOV3ip1CIS. (A) RT-qPCR following transfection of siRNA in SKOV3ip1CIS cells. **(B)** colony formation assay, and (**C-D)** invasion ability following siRNA transfections in SKOV3ip1CIS cells. **(E)** Cell viability following siRNA transfections in SKOV31p1 cells. Cell viability was performed with and without CIS (2.5 μM). (**F-I**) Cell viability and invasion following siRNA transfections in OVCAR3 and SKOV3ip1 cells. Mean ± SEM is shown relative to NC-siRNA (*$p < 0.05$, **$p < 0.01$, ***$p < 0.001$, and ****$p < 0.0001$).

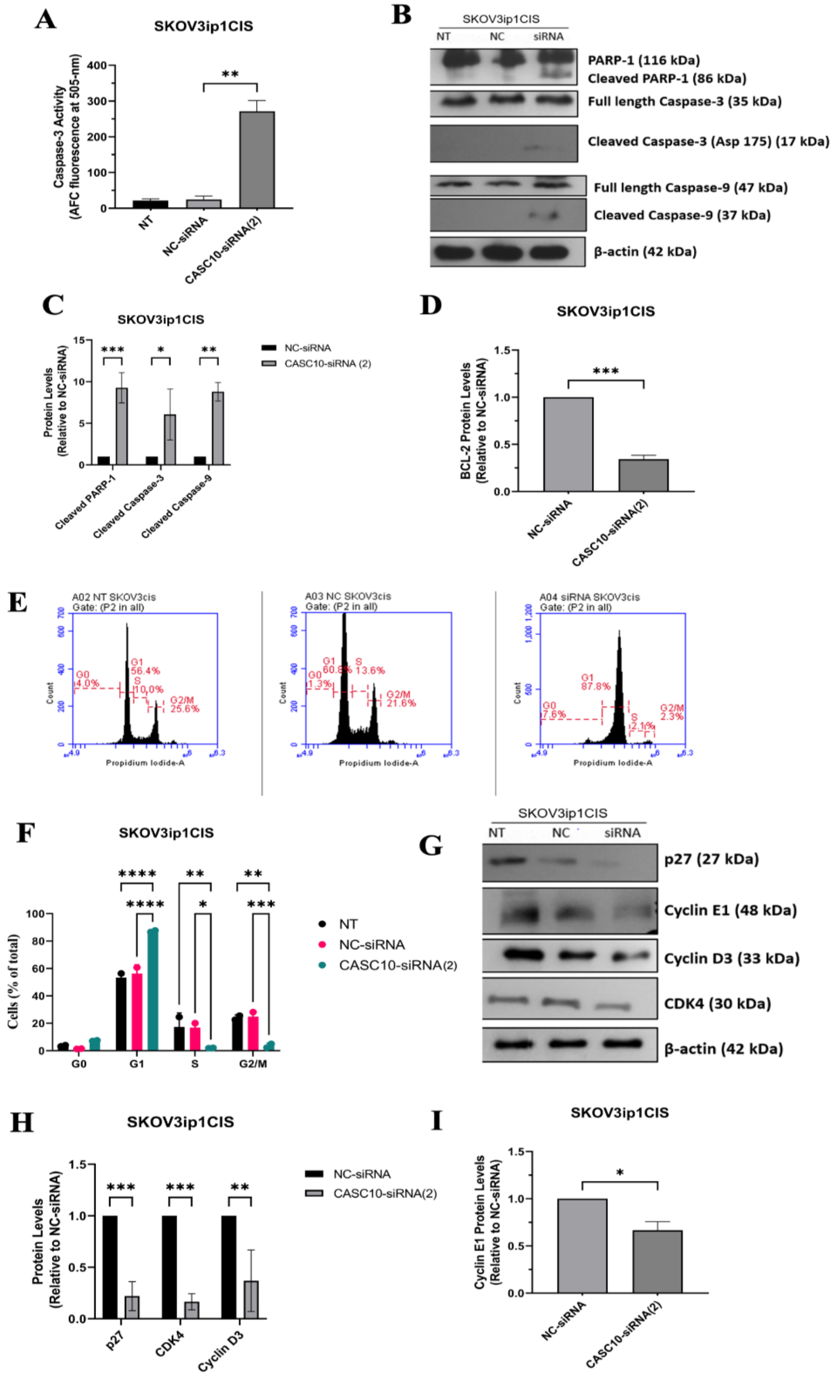

Figure S3. CASC10 siRNA-mediated knockdown Induced Apoptosis and Cell Cycle Arrest in SKOV3ip1CIS. SKOV3ip1CIS cells were transfected with 100 nM NC-siRNA or 100 nM CASC10-siRNA(2). **(A)** Caspase-3 fluorometric activity assay in SKOV3ip1CIS cells 72 hr after siRNA transfection. **(B)** Western blot analysis of apoptotic-related proteins. **(C-D)** Densitometric analysis of the band intensities shown in B. **(E)** Histogram showing cell cycle arrest at G0/G1 to S phase transition after CASC10-siRNA(2) transfection in SKOV3ip1CIS cells. **(F)** Quantification of the flow cytometry data shown in E. Values were expressed relative to NC-siRNA. **(G)** Western blot analysis of cell cycle-related proteins 48 hr after siRNA transfection. **(H-I)** Densitometric analysis of the band intensities shown in G. Mean ± SEM is shown relative to NC-siRNA (*$p < 0.05$, **$p < 0.01$, ***$p < 0.001$, and ****$p < 0.0001$).

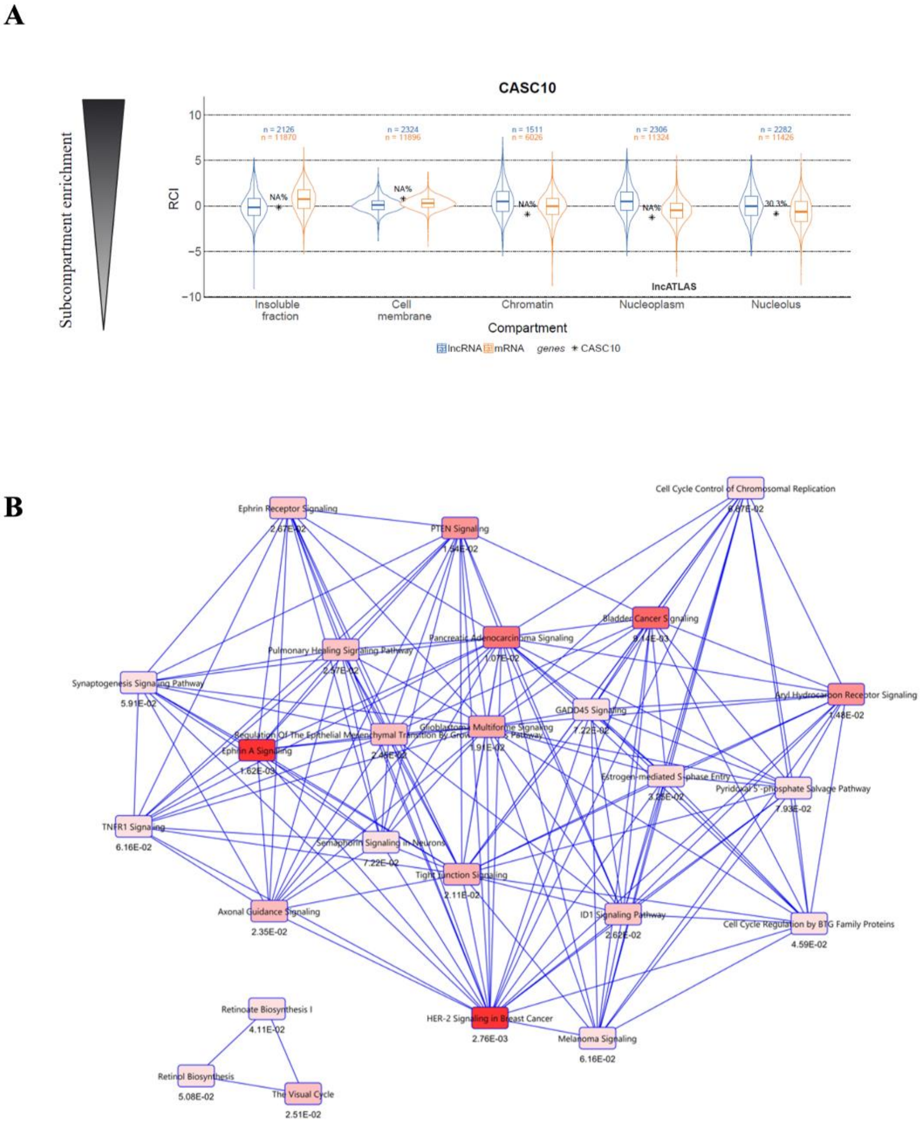

Figure S4. **(A)** Subcellular subcompartment analysis of CASC10 RNA levels in K562 cells. (**B**) Ingenuity Pathway Analysis (IPA) shows the additional top 25 canonical pathway interactions following siRNA-mediated knockdown CASC10 in OVCAR3CIS cells.

Table S1. List of the antibodies used for western blot analysis.

Primary Antibodies	Company	Species	Dilution
Caspase-9	Cell Signaling (9502)	Rabbit	1:500
Caspase-3	Cell Signaling (9665)	Rabbit	1:500
Cleaved Caspase-3	Cell Signaling (9664)	Rabbit	1:250
Cleaved Caspase-9	Cell Signaling (20750)	Rabbit	1:250
PARP-1	Cell Signaling (46D11)	Rabbit	1:1000
BCL-2	Abcam (7382)	Mouse	1:1000
CDK4	Cell Signaling (D9G3D)	Rabbit	1:500
Cyclin E1	Cell Signaling (HE12)	Mouse	1:1000
p27/Kip1	Cell Signaling (D69C12)	Rabbit	1:500
β-actin	Sigma	Mouse	1:10000

Table S2. Concentrations of cisplatin inhibiting 50% of cell viability. Incubation with cisplatin: 72-hr followed by Alamar Blue assay.

**Cell line**	**IC_{50} cisplatin (μM)**
A2780	0.5-0.8
A2780CP20	20-30
A2780CIS	4.3
OV90	3.5
OV90CIS	7.8
OVCAR3	2.8
OVCAR3CIS	7.5
SKOV3IP1	3.9
SKOV3IP1CIS	7.3

Table S3. List of the 414 differentially abundant RNA transcripts in cisplatin-sensitive vs. cisplatin-resistant HGSOC cells.

Gene Name	OV90log2FC (Resistant/Sensitive)	OVCAR3log2FC (Resistant/Sensitive)
**BCAT1**	2.849	4.562
**SH3BP4**	3.63	3.794
**NABP1**	-2.426	-5.027
**FGF2**	-5.443	-2.098
**STARD4**	-4.152	-3.39
**RGS2**	2.609	4.942
**ID4**	-6.583	-0.976
**PCYT1B**	-6.865	-0.760
**THBS1**	3.589	4.061
**LMO7**	5.368	2.285
**TENM3**	-2.439	-5.270
**AKAP12**	3.951	3.783
**SYTL2**	-3.189	-4.547
**CYP1B1**	3.069	4.801
**F2RL1**	3.652	4.226
**ATP11B**	-3.213	-4.713
**OGFRL1**	-3.043	-4.917
**SLC39A8**	-3.54	-4.474
**TNFAIP2**	6.296	1.946
**CRABP2**	-6.656	-1.622
**PAX9**	-5.501	-2.779
**NLGN1**	-3.428	-5.05
**ANKRD1**	-2.663	-5.829
**PDE1A**	0.964	7.528
**QPRT**	5.511	3.016
**TMEM30B**	-6.499	-2.105
**GDF15**	4.966	3.659
**SP3P**	-3.396	-5.275
**ITM2B**	4.733	3.947
**CA2**	6.825	1.868
**PCDH17**	-4.544	-4.152
**BRINP3**	-6.77	-1.955
**AKAP6**	-4.71	-4.035
**ANTXR2**	7.893	0.904
**MMP16**	-6.169	-2.687
**NRP1**	7.714	1.173
**A2M**	7.737	1.177

**EFEMP1**	8.179	0.765
**ABCC2**	7.886	1.073
**ZDBF2**	-4.461	-4.553
**PRSS23**	6.641	2.391
**MUC15**	-6.231	-2.856
**ARL4A**	-3.648	-5.454
**SLIT2**	-7.061	-2.093
**MID1**	-3.178	-6.01
**FAM198B**	4.885	4.342
**CCL26**	8.556	0.711
**CDH6**	8.573	0.742
**FAS**	-5.727	-3.592
**PROM1**	7.886	1.436
**LIPG**	3.67	5.678
**NAALAD2**	2.989	6.403
**LIN28A**	1.378	8.027
**MMRN1**	0.752	8.664
**HIST1H2BH**	0.755	8.683
**RBM47**	6.572	2.887
**SLC7A7**	7.533	1.93
**FGG**	8.522	1.004
**ZFP42**	0.876	8.785
**GLDC**	-6.067	-3.595
**NRK**	0.705	9.013
**JAG1**	8.212	1.523
**ISL1**	-4.549	-5.2
**LAMC2**	8.494	1.367
**NID1**	5.37	4.504
**RP3-332B22.1**	-6.373	-3.508
**FRY**	-5.233	-4.715
**ZPLD1**	1.86	8.16
**LHX8**	1.083	8.985
**MSX1**	1.8	8.323
**CD24**	8.619	1.508
**ADGRL2**	-4.868	-5.26
**POU1F1**	-6.368	-3.783
**LAMA2**	-4.911	-5.244
**RP11-115D19.1**	1.062	9.22
**MBNL2**	6.276	4.028
**ADAM28**	8.719	1.589

**CRISPLD1**	-6.566	-3.755
**TM4SF1**	8.555	1.792
**C3orf58**	-5.97	-4.393
**AARD**	-6.38	-3.992
**C1S**	6.658	3.802
**RHOBTB1**	3.937	6.563
**SULF1**	-6.791	-3.781
**HIST1H3G**	1.632	8.958
**ACTA2**	-7.269	-3.338
**SEMA3D**	-6.206	-4.422
**CLDN16**	-5.611	-5.292
**SPP1**	7.785	3.136
**GRID2**	-5.303	-5.68
**ARID5B**	3.283	7.713
**ANK3**	3.591	7.446
**IQGAP2**	6.336	4.8
**GNAT3**	-7.125	-4.09
**HDAC9**	3.747	7.556
**TOX**	-6.581	-4.901
**MMP3**	-6.707	-4.834
**VGLL3**	-6.179	-5.417
**RGS1**	2.701	8.926
**GPRIN3**	2.905	8.771
**MLIP**	-6.497	-5.213
**SIM1**	-6.263	-5.48
**SDPR**	-6.553	-5.299
**NRG1**	-6.513	-5.384
**BICC1**	4.73	7.199
**SYNPO2**	-6.801	-5.135
**SEMA3E**	-7.43	-4.543
**SLC8A1**	3.705	8.342
**TENM2**	-7.826	-4.225
**HIST1H3C**	8.647	3.475
**TMEM2**	5.24	7.101
**AMIGO2**	3.685	8.893
**NTS**	6.103	6.532
**WDR72**	8.587	4.104
**NUPR1**	6.008	6.844
**CAV2**	8.594	4.388
**PDGFRA**	8.246	4.799
**TGFB2**	5.758	7.377
**HIST1H2BE**	5.774	7.457

**SATB1**	4.759	8.473
**CARD16**	4.871	8.633
**FUCA2**	8.236	5.388
**MEST**	5.238	8.612
**HSPA1A**	7.348	6.879
**HIST1H2BI**	6.462	8.298
**UNC13C**	7.1	8.031
**HIST1H2AL**	7.753	7.459
**ZNF532**	7.147	8.119
**TES**	7.492	7.874
**HIST1H3I**	7.912	7.533
**HIST1H4D**	7.189	8.266
**HIST1H1B**	7.685	8.058
**DKK1**	7.451	8.426
**FTH1**	2.155	1.679
**PNMAL1**	-3.203	-0.632
**SACS**	-2.808	-1.027
**MAP1A**	2.177	1.674
**ARRDC3**	-1.682	-2.252
**SESN3**	-0.533	-3.407
**ARL6IP5**	-2.926	-1.04
**KLF3**	2.697	1.272
**TMCO3**	2.469	1.514
**ABRACL**	-2.029	-1.963
**CASK**	2.897	1.102
**CRIM1**	-2.361	-1.655
**GJA1**	-3.285	-0.789
**SORT1**	2.224	1.856
**ADAM15**	-3.681	-0.428
**FAM234B**	-2.457	-1.682
**U1**	-3.686	-0.496
**HMGB1P6**	-3.098	-1.088
**OCLN**	-0.438	-3.757
**HSPA4L**	-2.824	-1.39
**SPRY4**	2.373	1.907
**EMP3**	2.442	1.88
**CMBL**	3.615	0.71
**ANKRD10**	2.715	1.7
**SLC40A1**	3.524	0.932
**DDAH1**	-3.514	-0.965

**TNFRSF19**	-2.188	-2.369
**KLHL5**	1.834	2.727
**SLC9A7**	-0.966	-3.632
**CCDC138**	-2.347	-2.258
**SSX2IP**	-1.997	-2.616
**BCHE**	-3.329	-1.31
**HSPG2**	3.957	0.747
**PLAUR**	3.63	1.076
**MEIS2**	2.529	2.209
**PIK3CA**	-2.523	-2.357
**PSD3**	-2.396	-2.506
**TTC39A**	-3.818	-1.105
**ERRFI1**	3.773	1.328
**GCAT**	-3.073	-2.125
**EMP1**	-4.032	-1.205
**LIF**	-3.039	-2.24
**SLC12A6**	-2.707	-2.612
**MYO5A**	-3.998	-1.396
**IRS1**	4.252	1.218
**CFL2**	-3.496	-1.976
**USP53**	-1.397	-4.087
**MID1IP1**	3.254	2.232
**HEG1**	4.39	1.12
**NUP210**	-3.884	-1.669
**FTL**	4.429	1.148
**INSIG2**	2.282	3.31
**ATP11A**	4.262	1.333
**ARMCX6**	-2.951	-2.659
**KCNJ2**	-0.944	-4.697
**KITLG**	4.409	1.269
**TBC1D32**	-3.796	-1.935
**ACSL1**	-4.365	-1.368
**CITED2**	-2.153	-3.59
**LGR4**	3.476	2.299
**PCDH9**	-2.982	-2.859
**PCGF5**	-0.707	-5.141
**APOC1**	4.111	1.749
**KANK1**	-0.771	-5.112
**ADGRG6**	-0.969	-4.949
**ARHGAP28**	-4.771	-1.238
**PAX6**	-1.634	-4.429
**ABCA1**	1.447	4.634

**CPED1**	0.989	5.124
**PLAU**	4.736	1.385
**ABCA3**	-5.623	-0.53
**EYA4**	-5.638	-0.581
**CFH**	-3.3	-2.926
**TOM1L2**	-3.066	-3.174
**ARAP2**	-1.707	-4.549
**AMOT**	4.067	2.21
**IL1RAP**	-1.151	-5.142
**TSPAN8**	5.502	0.827
**CALCRL**	-5.567	-0.867
**PBX1**	4.161	2.288
**CASC10**	-6.033	-0.497
**HOXB9**	-1.628	-4.934
**MTUS1**	2.154	4.438
**FLRT3**	-1.312	-5.297
**SIX1**	-5.996	-0.722
**OXCT1**	-5.918	-0.829
**MPDZ**	-5.772	-0.996
**DNAJC22**	5.946	0.843
**GNAI1**	-1.418	-5.373
**HIC2**	5.971	0.861
**RGS5**	6.339	0.575
**PCDH7**	-1.697	-5.227
**ADAMTS3**	-6.244	-0.715
**NFKBIZ**	4.895	2.12
**SNRPN**	-6.44	-0.589
**ELOVL4**	-4.846	-2.184
**VCAN**	1.392	5.639
**MET**	4.825	2.207
**CDH12**	6.2	0.865
**SLC7A2**	1.712	5.473
**LRP1B**	-2.117	-5.17
**APOE**	5.742	1.55
**F2R**	3.526	3.801
**NEFH**	-6.276	-1.054
**RNLS**	-2.753	-4.604
**PLB1**	5.712	1.654
**XK**	-2.238	-5.142
**RUNX2**	0.686	6.85
**TP63**	-4.481	-3.09
**PAG1**	4.002	3.577
**ATP8B1**	5.935	1.666

**TOR4A**	6.637	0.965
**TDRP**	-6.636	-1.074
**RERG**	3.067	4.657
**HMCN1**	5.949	1.803
**SULF2**	5.871	1.882
**SCIN**	-2.704	-5.048
**C10orf10**	5.244	2.526
**SOSTDC1**	3.635	4.183
**MGAT3**	4.748	3.083
**C2orf72**	5.504	2.334
**TMCC3**	5.595	2.255
**S1PR1**	0.768	7.114
**PALD1**	-7.191	-0.707
**MCOLN2**	-6.298	-1.61
**PROCR**	2.216	5.769
**CCDC74A**	-6.246	-1.759
**CFP**	6.971	1.163
**PDGFC**	-3.449	-4.711
**RNF144B**	-6.53	-1.645
**VIL1**	6.498	1.683
**NYNRIN**	4.242	3.95
**TMEM178A**	-4.283	-3.915
**VTN**	7.014	1.203
**TGFBR3**	3.754	4.464
**ADGRB3**	-6.687	-1.532
**ZNF415**	4.641	3.591
**ZNF704**	2.274	5.973
**SULT1A1**	4.823	3.435
**CACNA1H**	-3.147	-5.151
**ESPN**	-4.683	-3.694
**NUDT11**	-6.273	-2.126
**LPAR1**	-3.441	-5.001
**CCDC74B**	-6.883	-1.585
**CDH10**	-7.619	-0.872
**COL17A1**	6.168	2.348
**NR3C1**	7.019	1.502
**FAM184A**	-3.73	-4.797
**RGL1**	4.965	3.578
**KIF1A**	-7.199	-1.433
**HPGD**	7.569	1.277
**SLITRK6**	-6.079	-2.77
**TMEM184A**	-3.868	-4.986

**MMP14**	7.021	1.885
**LRRC17**	7.498	1.424
**ABCB1**	-4.17	-4.788
**SH2D5**	-6.523	-2.51
**CFTR**	7.291	1.814
**NRXN1**	-5.791	-3.448
**PIK3C2G**	7.315	2.103
**PPP1R1C**	4.773	4.724
**FAT3**	7.963	1.588
**TRPM3**	1.672	7.897
**SLC5A5**	7.696	1.898
**RP1-261D10.2**	-5.256	-4.339
**IGFBP4**	6.89	2.723
**SYNE4**	-5.479	-4.176
**SLC16A14**	-6.305	-3.386
**TFEC**	5.917	3.817
**EPAS1**	6.717	3.019
**FBXL7**	-7.228	-2.513
**NLGN4X**	0.932	8.871
**LINC01351**	-6.664	-3.153
**PCLO**	-6.521	-3.312
**BST2**	3.65	6.185
**GRIK1**	2.112	7.739
**LSAMP**	8.005	1.852
**DISC1FP1**	-5.6	-4.293
**NPNT**	7.795	2.108
**HIST1H4L**	8.824	1.158
**PTGIS**	7.141	2.896
**OTOA**	1.426	8.664
**KCNT2**	-5.007	-5.086
**ARL10**	3.691	6.456
**PPARGC1A**	8.514	1.695
**DNAJC6**	-4.159	-6.053
**MXRA8**	6.126	4.138
**RUNX1**	8.628	1.643
**PDLIM3**	-4.761	-5.558
**ONECUT2**	8.279	2.046
**EPHA5**	-7.009	-3.321
**HIST1H2BB**	5.909	4.44
**IGFBP3**	-7.315	-3.037

**CCDC110**	-5.467	-4.905
**FZD8**	4.698	5.787
**SLITRK5**	-7.027	-3.462
**VWDE**	-5.336	-5.231
**TRPV2**	6.268	4.389
**TBX15**	-6.049	-4.642
**CFAP47**	-5.781	-4.912
**LRG1**	7.975	2.723
**EPDR1**	-6.742	-3.985
**TPH2**	-2.766	-8.037
**HIST1H3J**	7.052	3.78
**TRHDE**	-3.021	-7.824
**PTPRD**	3.016	7.876
**MYEOV**	8.133	2.771
**ZNF788**	1.749	9.172
**VEGFC**	-6.108	-4.837
**RASSF9**	5.881	5.08
**CAV1**	5.354	5.612
**USP44**	7.722	3.286
**CDCP1**	8.535	2.478
**TNS3**	7.512	3.554
**FAM110C**	-7.228	-3.839
**SNTG1**	-6.821	-4.248
**FMN1**	-6.023	-5.088
**FAM111B**	6.931	4.201
**GS1-600G8.3**	-6.687	-4.473
**PDE4B**	-6.284	-5.017
**SPAG6**	-7.059	-4.258
**SLC6A15**	-5.882	-5.452
**DCDC1**	-6.34	-5.004
**ELF3**	7.918	3.49
**GAS1**	-6.48	-5.013
**NID2**	5.152	6.351
**EOMES**	3.634	7.871
**INPP1**	8.383	3.132
**GSG1**	-6.509	-5.125
**PRRX1**	-7.367	-4.314
**SV2A**	3.705	7.979
**SATB2**	-6.778	-4.926
**TMEM207**	-5.756	-5.968
**ZNF521**	2.909	8.816
**LIN7A**	-7.011	-4.716

**MAGI2-AS3**	-7.043	-4.79
**KIFC3**	7.583	4.277
**AF121898.3**	-6.78	-5.114
**ADAMTS9**	8.532	3.404
**GALNT13**	-7.169	-4.782
**ACSS3**	-6.822	-5.131
**MUSK**	-6.5	-5.476
**SLC9A9**	-6.374	-5.631
**CASP4**	5.813	6.255
**CPA4**	7.797	4.435
**RP11-865I6.2**	-7.152	-5.089
**TMEFF2**	-8.146	-4.125
**PRKG1**	5.656	6.652
**RP11-255H23.2**	3.494	8.884
**NRG3**	9.075	3.33
**ZNF595**	4.361	8.078
**SLC44A5**	7.692	4.886
**AGMO**	5.557	7.102
**PDGFD**	5.224	7.436
**SOX9**	8.431	4.234
**ELFN1**	4.608	8.097
**MGST2**	8.215	4.519
**TGFB2-AS1**	5.167	7.593
**RP11-2E11.6**	4.689	8.176
**PTPRQ**	-6.108	-6.981
**OLFML3**	4.882	8.284
**PLBD1**	5.84	7.566
**AVPR1A**	8.135	5.296
**ZNF841**	7.715	5.867
**ESRRG**	5.439	8.144
**ZNF681**	5.459	8.4
**FLNC**	5.98	7.962
**HSPB1**	6.297	7.656
**S100A11**	7.449	6.726
**TMEM156**	8.629	5.626
**GPX1**	7.616	6.787

**TGFB1I1**	7.35	7.098
**MDFIC**	8.361	6.556
**ALDH2**	7.644	7.304
**HIST1H4A**	8.143	6.869
**ZNF320**	6.905	8.371
**ZNF468**	8.079	7.213
**C14orf169**	7.129	8.282
**HIST1H3A**	8.105	7.314
**ZNF718**	6.764	8.705

Table S4. CASC10 downstream signaling pathways generated by IPA.

Network	Diseases and Function	Molecules
1	• Cell Death and Survival<br>• Cellular Movement<br>• Developmental Disorders	TPPP, PYM1, CD151, MAGOH, NRSN1, GLYR1, TMEM64, CELF1, PTBP1, MYC, SERINC3, Cyclin D, SNHG3, EPHA4, CDC42, MERTK, CDK4, PDLIM3, CDCA7L, TNF, CNN1, CDC42EP4, AQP3, TRIM5, FSH, ARHGAP1, IFRD2, WT1, SRY, SLC20A1, EFNA1, TNFRSF6B, TMBIM6, ETV5
2	• Cell Cycle and Chromosomal Regulation<br>• Cancer and Cell Survival	PCDH7, COL4A1, SMAD6, ANXA9, COL6A2, COL4A2, COL5A1, NFIB, HMGA1, ESTROGEN RECEPTOR, NORAD, EGLN2, ABCA1, TIMP2, KRT18, PDGFA, TGFBR2, FGFR3, CRNDE, LTB, SP1, RHOB, LIPA, NCOR2, GADD45B, , CR2, RB1, NOX4, SIN3A, CDC37, KDM5A, SIN3B, MORF4L1, MORF4L2, MRFAP1

A

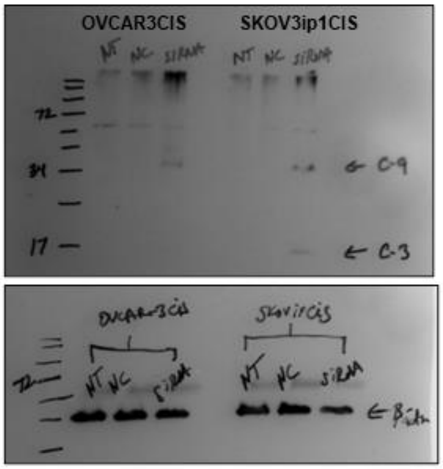
OVCAR3CIS
SKOV3ip1CIS
NT NC siRNA
NT NC siRNA
72
34
17
C-9
C-3
NT NC siRNA
NT NC siRNA

B

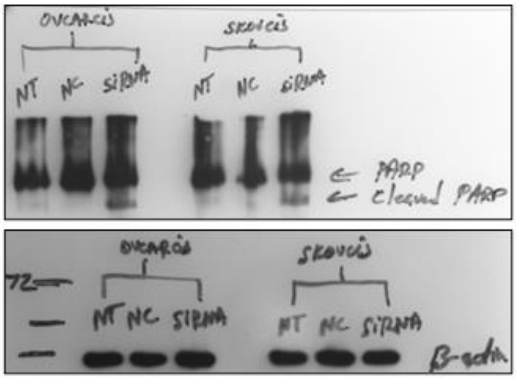
NT NC siRNA
NT NC siRNA
PARP
Cleaved PARP
72
NT NC siRNA
NT NC siRNA
β-actin

C

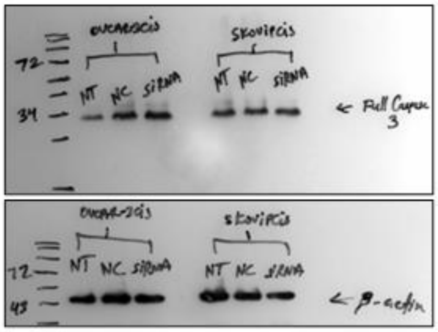
72
34
NT NC siRNA
NT NC siRNA
Full Caspase 3
NT NC siRNA
NT NC siRNA
72
43
β-actin

D

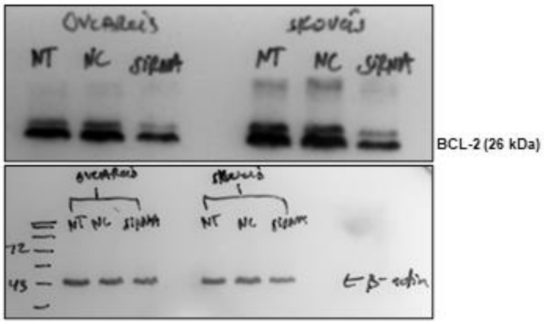
NT NC siRNA
NT NC siRNA
BCL-2 (26 kDa)
72
43
β-actin

E

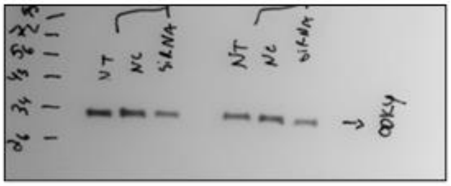
NT NC siRNA
NT NC siRNA

F

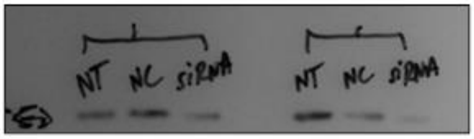
NT NC siRNA
NT NC siRNA

G

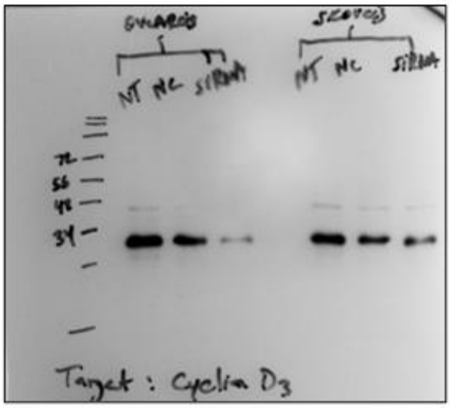
NT NC siRNA
NT NC siRNA
72
56
43
34
Target : Cyclin D3

H

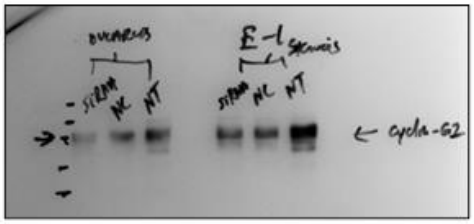
siRNA NC NT
siRNA NC NT
cyclin-G2

Figure S5. Western blot images. Protein bands and molecular markers are shown for (A) Cleaved Caspase-3 and Cleaved Caspase-9, (B) PARP and Cleaved-PARP, (C) Full Caspase-3, and (D) BCL-2. A-D corresponds to Figure 4B and Figure S4B. (E) CDK4, (F) p27, (G) Cyclin D3, (H) Cyclin E1. E-H corresponds to Figure 4G and Figure S4G.
